# Barriers and Facilitators Influencing Real-time and Digital-Based Reporting of Adverse Drug Reactions by Community Pharmacists: Qualitative Study Using the Task-Technology Fit Framework

**DOI:** 10.2196/40597

**Published:** 2022-10-11

**Authors:** Joel Fossouo Tagne, Reginald Amin Yakob, Rachael Mcdonald, Nilmini Wickramasinghe

**Affiliations:** 1 Department of Health Science and Biostatistics School of Health Sciences Swinburne University of Technology Melbourne Australia; 2 MedTechVic Swinburne University of Technology Melbourne Australia; 3 Australian Association of Consultant Pharmacy Sydney Australia; 4 Department of Nursing and Allied Health, Occupational Therapy Swinburne University of Technology Melbourne Australia; 5 Iverson Health Innovation Research Institute Swinburne University of Technology Melbourne Australia; 6 Epworth Healthcare Melbourne Australia

**Keywords:** pharmacovigilance, adverse drug reaction, pharmacist, Task-Technology Fit, digital health

## Abstract

**Background:**

Medication use can result in adverse drug reactions (ADRs) that cause increased morbidity and health care consumption for patients and could potentially be fatal. Timely reporting of ADRs to regulators may contribute to patient safety by facilitating information gathering on drug safety data. Currently, little is known about how community pharmacists (CPs) monitor, handle, and report ADRs in Australia.

**Objective:**

This study aimed to identify perceived barriers to and facilitators of ADR reporting by CPs in Australia and suggest digital interventions.

**Methods:**

A qualitative study with individual interviews was conducted with CPs working across Victoria, Australia, between April 2022 and May 2022. A semistructured interview guide was used to identify perceived barriers to and facilitators of ADR reporting among CPs. The data were analyzed using thematic analysis. We constructed themes from the CP-reported barriers and facilitators. The themes were subsequently aligned with the Task-Technology Fit framework.

**Results:**

A total of 12 CPs were interviewed. Identified barriers were lack of knowledge of both the ADR reporting process and ADR reporting systems, time constraints, lack of financial incentives, lack of organizational support for ADR reporting, inadequate IT systems, and preference to refer consumers to physicians. The proposed facilitators of ADR reporting included enhancing CPs knowledge and awareness of ADRs, financial incentives for ADR reporting, workflow-integrated ADR reporting technology systems, feedback provision to CPs on the reported ADRs, and promoting consumer ADR reporting.

**Conclusions:**

Barriers to and facilitators of ADR reporting spanned both the task and technology aspects of the Task-Technology Fit model. Addressing the identified barriers to ADR reporting and providing workplace technologies that support ADR reporting may improve ADR reporting by CPs. Further investigations to observe ADR handling and reporting within community pharmacies can enhance patient safety by increasing ADR reporting by CPs.

## Introduction

### Background

Pharmacovigilance (PV) is defined by the World Health Organization as the “science and activities relating to the detection, assessment, understanding, and prevention of adverse events or any other drug-related problem.” The collection and reporting of safety data commence from the initial stages of drug development, throughout the clinical trials, and continue once a medicine is registered and marketed around the world, that is, postmarketing surveillance [1,2]. Medication use can result in adverse drug reactions (ADRs) that cause increased morbidity and health care consumption for patients and could potentially be fatal. In Australia, approximately 7.2% to 11% of hospital admissions are ADR related [3]. Globally, studies have reported ADR-related hospital admissions ranging from 3.6% to 15.6% [4,5]. The health care costs of ADRs may be high owing to complexities associated with ADR treatment, with a reported mean length of hospital stay increasing from 8 to 20 days [6]. In Australia, the annual cost of medication-related problems was reported as Aus $1.4 billion (US $900,207) in the Pharmaceutical Society of Australia’s medication safety report (2019) [7].

In 2017, the Therapeutic Goods Administration (TGA) of Australia received approximately 18,600 reports of adverse drug events [8]. Among the ADRs reported to the TGA, approximately 53.75% (n=9998) were from sponsors, that is, marketing authorization holders; 18.5% (n=3441) from state and territory health departments; 10.1% (n=1879) from hospitals and hospital pharmacists; 6.45% (n=1201) from consumers, that is, the public; 6.29% (n=1170) from community pharmacists (CPs); 3.11% (n=579) from general practitioners; and 1.93% (n=359) from other sources [8]. Increased participation of CPs in ADR reporting is important, as CPs are usually the first point of contact regarding medication-related issues and the most frequently visited health care professionals (HCPs) in Australia [9].

As of May 9, 2022, there were 5822 community pharmacies across Australia and, on average, a consumer is estimated to visit a community pharmacy approximately 18 times each year in metropolitan, rural, and remote locations [10,11]. In metropolitan cities, 97% of the consumers are within 2.5 km of a pharmacy and in regional or remote areas, 65% of the people are within 2.5 km of a pharmacy [10]. Annually, more than 462 million patients visit community pharmacies [11,12]. CPs are the most frequently accessed and visited of HCPs, with almost 218.3 million prescriptions dispensed through the Australian Pharmaceutical Benefits Scheme in 2021 [10]. As a general aspect of community pharmacy practice, CPs interact with and may counsel consumers about their medications, adverse effects, or other medicine-related issues that the consumer may have experienced [7,13].

In Australia, CPs are expected, as part of their training, to possess medication counseling skills and professional knowledge on topics including pathophysiology, therapeutics, disease prevention, management, and treatment within their scope of practice [13,14]. Patients or consumers can visit CPs without needing an appointment, offering professional health management services that can complement the services of other health professionals, for example, CPs triage consumers and refer them to other health professionals, as necessary. This may decrease the public’s demand for services in congested emergency departments and medical clinics [14]. Such support is especially important during a health crisis such as the COVID-19 pandemic. Therefore, in Australia, CPs are ideally placed to provide a person-centered solution to support the public regarding their health concerns [7,14].

As noted by Li et al [9,15], there is very little literature on the perspectives of CPs on ADR reporting in Australia. The barriers to ADR reporting by CPs in Australia have not been extensively investigated, and to our knowledge, only 1 study concerning the perspectives and knowledge of reporting of ADR by Australian CPs has been identified [9,15]. In the study by Li et al [9], 43% (n=101) of the respondents agreed that a lack of time within their professional practice limited their reporting of ADRs, and 65% (n=150) agreed that remuneration would encourage them to report ADRs. The integration of autopopulation features within the dispensing software was identified as an efficient way to facilitate ADR reporting by CPs [9]. Such findings are also consistent with those of studies in other countries [16-18].

### Leveraging Technology in ADR Reporting

The incorporation of technology into health care provisions is currently prevalent [19]. As an example, the COVID-19 pandemic–related lockdowns acted as a *catalyst* that accelerated digital health transformation through the introduction of telehealth and electronic prescribing [11]. To maximize the benefits of incorporating technology into the practice of health professionals (including CPs), the service provided by the technology should reasonably match the practice requirements of the clinician [11,20]. Therefore, it is also necessary to understand the factors that may affect an end user’s workflow tasks and information requirements [20]. A 2020 systematic review of interventions to improve ADR reporting concluded that there was a lack of consideration of theoretical frameworks in the design of interventions [16]. Furthermore, there is also a lack of end-user input (ie, HCPs) in the design of ADR reporting systems, with only the needs of regulatory agencies taken into account [21].

Knowledge gaps exist regarding ADR reporting by CPs in Australia and the need for IT support within the ADR reporting domain. As such, research is needed to better understand the factors influencing ADR reporting within the CPs workflow and related digital intervention needs. This study aimed to identify the barriers to and facilitators of ADR reporting by CPs, which may inform the design and development of tailored technological interventions.

## Methods

### Overview

A qualitative study with semistructured interviews was conducted with CPs (N=12) working in community pharmacies across Victoria, Australia, between April 2022 and May 2022. To understand knowledge constructed through a pharmacist practice lens, the underlying epistemology stemmed from the social-constructivist paradigm. Purposive sampling was used to select eligible participants working in community pharmacies listed on the Pharmacy Guild of Australia and Health Direct website. Participants were invited by email to recommend other CPs for participation. Eligible participants were sampled according to the modified Monash (MM) category. The model measures rural remoteness and population size on a scale of MM1 to MM7, where MM1 is a major city and MM7 is very remote [22]. A qualitative study design was used to highlight the individual and system-related factors that influence ADR reporting among CPs. The classification of barriers and facilitators associated with ADR reporting was mapped to 2 target domains of a sociotechnical framework.

Other studies have discussed interventions to improve ADR reporting among health professionals in different countries [15,16]. However, it is important to note, the rationale for selecting these interventions may not have a theoretical base [16,23]. Therefore, it is necessary to understand the ADR reporting behavior of CPs using a well-defined theoretical approach [16]. Interventions are not uniform, that is, an intervention applied in one setting may not be appropriate for another health setting, and there is a need for stronger evidence that guides the selection of relevant and comprehensive interventions [24].

### Theoretical Model

**Figure 1:**
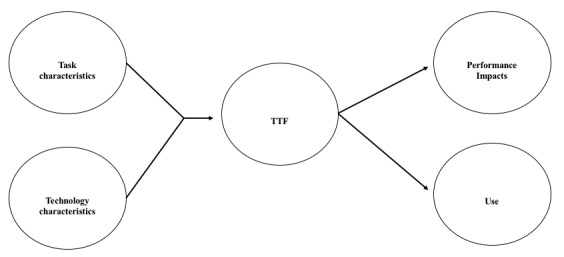
Task-Technology Fit (TTF) theory [27].

Researchers have used technology adoption models and diffusion theories to build a foundation for studies to understand innovation adoption and diffusion [25]. Technology has become a fundamental aspect of our society and is being embedded within every aspect of health care delivery, with a shift toward a digital health care ecosystem [11]. HCPs’ behavior in reporting ADRs can be influenced by different factors, including individual characteristics and those that involve the external environment [26]. As such, the Task-Technology Fit (TTF) theory provides a theoretical lens and guidance for research (Figure 1) [26,27].

Task characteristics refer to the attributes of a task that can be executed using information communication technologies to satisfy work practice needs (eg, dispensing a prescription or ADR reporting). Tasks can vary in several dimensions, including task nonroutineness, task interdependence, and time criticality. The users’ workflow and environment are also key considerations when assessing the “Fit” [27].

Technology characteristics refer to the tools used by individuals to carry out their tasks. Aspects of technology tools may influence technology use and user perceptions. The TTF model considers the importance of fitting the functionality and attributes of technology to the demands imposed by individual needs. These tools can either be hardware or software [28].

The TTF model has been applied in health care settings where businesses require technology solutions [29]. Because this research also sought to explore strategies to implement innovative technologies to facilitate ADR reporting, the TTF model offered guidance when developing the semistructured interview questions and categorizing identified themes [11].

### Data Collection

All participants were asked the same semistructured questions, and appropriate probing questions were used when necessary to draw out information for the study from each respondent. They were also given the freedom to express additional views on topics discussed at the end of each interview session. Participants were informed about the purpose of the study, which was not to audit their practice but to understand their perceptions of the problems of spontaneous ADR reporting and ways to improve the current system in place. The research team developed an interview guide during several rounds of discussions. The questions were categorized according to the TTF model to cover the relevant domains. A total of 2 pilot interviews with CPs were conducted to test the interview guide for comprehensibility and clarity. Participants provided feedback on the interview guide, and after minor adjustments, a final version was made. Each interview session lasted approximately 20 to 50 minutes and was conducted by the researcher at a place and time convenient for the pharmacist, mostly via a web-based video, using Microsoft Teams, or face to face in a private area within the premises where the pharmacist practiced. The interviews were audio recorded and transcribed verbatim, automatically, using the Otter.ai transcription service. The researcher then listened to the tapes and manually rechecked the transcripts line by line for accuracy and removed any identifying information. We continued to collect data until no new themes related to the research questions could be identified.

### Ethics Approval

Before conducting the interviews, all participants provided informed written consent to participate in the study and were advised that the information provided, although deidentified, could be used for publication. Participants’ demographic data were collected using a self-administered questionnaire attached to the consent form. All procedures were in accordance with Australia’s National Statement on Ethical Conduct in Human Research (2018). This study was approved by the Swinburne University of Technology Human Research Ethics Committee (reference 20214304-6249).

### Data Analysis

Thematic analysis began once the interviews were completed using NVivo (version 12; QSR International) software. Initially, open codes were generated inductively from participants’ descriptions of their experiences in reporting ADRs and the barriers to or facilitators of reporting. Following the initial coding of the transcripts, preliminary themes that captured information relevant to the research questions were generated. This process involved identifying patterns within the data, including recurring ideas, perspectives, and descriptions that depicted each participant’s context and perspective. The final analysis focused on the key themes constructed from the interviews and were subsequently mapped to the TTF model. Data concordance was verified by NW and RM, researchers with extensive experience in public and digital health research. Key themes were discussed with the research team that included JFT and RAY, clinicians with expertise in quality use of medicine and drug safety. The interviews concluded when no additional themes could be identified and mapped to our theoretical framework.

## Results

### Overview

After interviewing 12 participants, including 6 (50%) CPs from MM1 (metropolitan areas or major cities), 5 (42%) from MM2 (regional centers), and 1 (8%) from MM4 (medium rural town), interviews were concluded. No participants were interviewed from MM3 (large rural towns), MM5 (small rural towns), MM6 (remote communities), or MM7 (very remote communities) per the MM category described in Multimedia Appendix 1. The demographic characteristics of the participants are presented in Table 1.

**Table 1:** Demographic characteristics of the community pharmacists (N=12).

Demographics		Participants, n (%)^a^
**Sex**		
	Male	7 (58)
	Female	5 (42)
**Pharmacist age (years)**		
	20-25	—^b^
	26-35	10 (83)
	36-45	2 (17)
	46-55	—
	56-65	—
	66-75	—
**Employment status**		
	Supporting pharmacist	2 (16)
	Pharmacist in charge	5 (42)
	Manager	5 (42)
	Owner	—
**Pharmacist experience in community pharmacy (years)**		
	<1	—
	1-2	4 (33)
	2-4	—
	5-10	5 (42)
	>10	3 (25)
**Pharmacist education level**		
	Bachelor	1 (8)
	Honors	4 (33)
	Graduate certificate	1 (8)
	Graduate diploma	1 (8)
	Master	5 (42)
	PhD	—
**Pharmacy’s average number of prescriptions per day**		
	0-50	1 (8)
	51-150	3 (25)
	151-250	2 (17)
	251-350	3 (25)
	351-450	1 (8)
	451-550	—
	>550	1 (8)
	Not disclosed	1 (8)
**Number of hours working in community pharmacy per week**		
	<10	2 (17)
	11-20	—
	21-30	—
	31-40	4 (33)
	>40	6 (50)
**Pharmacy’s classification of rural rank^c^**		
	MM1^d^	6 (50)
	MM2	5 (42)
	MM3	—
	MM4	1 (8)
	MM5	—
	MM6	—
	MM7	—

^a^The sum of percentages may not be 100%, as the values were approximated to the nearest tenth.

^b^Not available.

^c^Refer Multimedia Appendix 1.

^d^MM: modified Monash.

All participants reported capabilities in identifying drug interactions or side effects during their daily practice; however, most were not accustomed to the conscious practice of PV, that is, ADR monitoring, handling, and reporting. Of the 12 participants, 2 (17%) had reported 1 ADR in the past 12 months, both relating to a COVID-19 vaccination, and 2 (17%) recalled completing at least one ADR report relating to medications over the past 5 to 10 years. Conversely, other participants (8/12, 66%) had never reported an ADR to a state or regulatory authority. Three major themes were identified in our results: (1) poor knowledge of PV, (2) low awareness of ADR reporting, and (3) work environment or resources influencing ADR reporting.

Overall, participants reported having little to no training in PV at the university or postuniversity level. All participants acknowledged that education could improve their awareness about ADRs and ADR reporting:

I don’t think I learned about it in university and I've been working since second year i.e. in community pharmacy. None of the pharmacists I've seen ever makes a reporting of adverse reaction.

CP3

I really don't think any of the pharmacists think about it to be honest, because it's not something you're trained to do, and you’re not incentivised to do it. It's not part of the workflow.

CP10

According to 17% (2/12) of reporting pharmacists with experience working outside the community pharmacy sector, lack of education and understanding of ADRs is a common factor:

In terms of looking at the undergraduate and registration year, I don't think it teaches much, in particular, understanding the differences between side effects, adverse drug events, and adverse drug reactions.

CP5

I had no idea what that word meant. When I was working in the pharma industry, I had all these SOPs to read on pharmacovigilance and I'm like, “what is that?” I hope it’s part of the syllabus now, because I think pharmacists should be at the forefront of pharmacovigilance.

CP11

Themes were divided into 2 broad categories, corresponding to the components of the TTF model. Perceived barriers to and facilitators of ADR reporting affected tasks and technology. These themes are discussed and illustrated using quotes (Textbox 1).

**Barriers and facilitators perceived by participants categorized into the Task-Technology Fit model.**

**Factors affecting the task**

- Barriers
  Lack of support (lack of time)
  Lack of financial incentive (low adverse drug reaction [ADR] priority)
  Referring consumer to corresponding physicians or hospital takes priority
- Facilitator
  Enhanced knowledge or awareness of pharmacovigilance and ADR reporting
  Environmental restructuring (financial incentive for ADR reporting and workplace support)
  Empower consumer reporting

**Factors affecting technology use**

- Barriers
  Low awareness or lacking knowledge of reporting systems
  Inadequate IT systems
  Fragmented reporting systems
- Facilitator
  Centralized or streamlined reporting systems
  User-friendly reporting systems (integrated within the clinician workflow; autopopulation features; efficient reporting forms; artificial intelligence)
  ADR reporting mobile apps
  Consumer follow-up and clinician feedback

### Barriers

Participants were asked about factors that negatively affected their willingness to report ADRs. All participants said they did not report the ADRs that they encountered in practice owing to either a lack of time or financial incentive, where workload pressure and not knowing how to access the reporting forms were identified as key drivers in the “lack of time” for reporting.

### Task

#### Lack of Support as a Driver of Time

The participants’ professional organizations and lack of supporting staff were key barriers to reporting ADRs. This was related to the effort and time needed to complete an ADR during or after clinical interaction with customers:

If I did e.g. sick certificate and I know it’s going to take 5 to 10 minutes and there are people constantly coming in and out dropping off scripts. When I come back to the scripts, what might have been a 10-minute wait before is now a half an hour wait. So, there are times when people [CPs] just send them away.

CP2

Unless you're doing it exactly at the time of the adverse event, it takes time. First of all you find time between your work to do it, then you have to recollect everything as accurately as you can, which also becomes more time consuming because you spend more time trying to remember what happened, because you can't do it at the time of the incident.

CP3

#### Lack of Financial Incentive as a Driver of ADR Nonreporting

Most participants expressed a lack of incentives in the form of financial rewards, stating that prescriptions are what brings money to the pharmacy:

So rightly or wrongly, the pharmacists focus is on getting to the next prescription or satisfying the customers.

CP4

I feel like there should be some kind of incentive like last year, with the 6th CPA [community pharmacy policy agreement] agreement.

CP10

#### Referring Patients to Corresponding Physicians

When discussing how the participants handled ADRs or adverse events in their daily practice, most participants considered ensuring safety as the initial priority and then notified their physician or referred them to the hospital as the default:

If there's an adverse reaction, you'd call the doctor to explain what's happened and do everything to see that the patient is fine. I was never encouraged or ever prompted to, “hey, this is a risk and that you need to report it” So yeah, there's probably a huge underreporting.

CP12

Well, if a customer comes in and says “something [medication] is giving me this side effect.” Then, I'll go through each medication and see what potentially could be causing it, then contact the prescriber and maybe switch over to a different one.

CP9

### Technology

#### Lack of Knowledge of Reporting Systems

The participants were asked about their familiarity with the PV and ADR reporting systems, and most stated that they would normally use Google search. This included both the reporting and nonreporting CPs:

When I say confusing, let's say, I want to report something online. I need to Google it, find out what organization it is and under what platform.

CP6

I know how to report vaccines’ adverse reaction, but regarding medications, not really. If someone came in with an ADR, then I'll have to Google and that would take a chunk i.e. [time] out of my day and I don’t want to report it to the wrong place.

CP3

#### Low Awareness of the Guild ADR Recording Module

The participants were asked if they had used the GuildCare professional service programs before. This was followed up with their familiarity with the built-in ADR recording module, that is, the first ADR reporting feature enabling CPs to report directly to the TGA in Australia. All participants recalled using the platform at some point within the practice to carry out professional services; however, none were aware of the ADR reporting feature:

I use GuildCare a little bit for HMRs [Home Medication Review] and medchecks i.e. [pharmacist medication reviews services] and no, this is the first I've ever heard of it. Well in my experience, I would say the first barrier is awareness, I’ve practiced for 13 years and I have never even known it existed.

CP7

Now we only use it for project-stop i.e. [national pseudoephedrine drug surveillance system] or Covid-19 RAT tests [rapid antigen test].So no, because I also worked for a year in New South Wales, there they also used GuildCare, I only did medchecks but I didn’t know about the adverse reaction was part of it.

CP1

#### Inadequate IT Systems

Participants emphasized the need for an adequate and user-friendly IT system that facilitates ADR reporting. For instance, functional fields that are easy and quick to access during a consultation. Participants also valued a single national system that facilitated information exchange with other relevant HCPs in primary or secondary care:

It takes like a long time, you have to create like different 10 accounts, then link them and just to answer five questions or some questionnaires won't allow me to specifically say, what the adverse reaction is. If I have to choose between five options and it's none of the five, then I'm going to have to choose the closest thing and just it doesn't feel right.

CP3

I know this sounds bad, it’s a lot of paperwork, like I said with COVID-19, we do a lot of reporting as a company which is fantastic. But the fact that sometimes when you have to go on a website, find the link, download the link, fill it out, submit it to this authority, then you have to go to another authority, which is on a completely different website, and then you get an email back which you have to follow up. It's not very streamlined, and I'm not going to lie, It’s hard work.

CP9

### Facilitators

CPs were asked what would facilitate the ADR reporting process; the interviewees highlighted the importance of feedback from authorized agencies, the inclusion of topics related to ADR reporting in the pharmacy curriculum, improvements in the training programs, continuing professional development, financial incentives, and integrating innovative information systems within their workflow.

### Task

#### Enhanced Knowledge and Awareness of ADRs

Participants recalled briefly learning about PV during university, and were not aware of any professional training modules or education campaigns:

Probably more awareness on it, make it part of the actual pharmacy school and part of the Intern Training Programs so it becomes a routine thing. If they wanted to start to bring it up right now you have to run basically an awareness campaign so it's something that you do remember.

CP2

Yeah, awareness and education, modules, maybe push the managers go through it with the team on how to report and show them the system.

CP7

#### Environmental Restructuring (Financial Incentive for ADR Reporting)

All participants agreed that if ADR reporting could be incentivized, that could encourage more reporting:

You know, this sounds really bad. But I'm sure if there were incentives for people would do it.

CP9

I said the boss's aim is you can't just stand there and do something that's not going to bring in the money whilst people take their business elsewhere. So even if it was just a tokenistic amount of money to recognize that it takes time to fill in these forms, would be an enabler.

CP4

#### Empower Consumers Reporting

Encouraging consumers to become more active in ADR reporting was highly regarded by the participants. Furthermore, this was also regarded as a positive way to reduce workload pressure:

If the consumers know this as well or if there's a way to give them a pamphlet and say, “just report it, it's important that you do it, it helps in the future.” So maybe if there's a system like that, it might be taking the pressure off us but still able to get the information across.

CP5

I think patients can be empowered more to report then they don't have to go to the healthcare professional. So, education and messaging to patients to take a bit of ownership on their medications, their adverse events and reporting it to either the TGA or to the company.

CP11

### Technology

#### Centralized or Streamlined Reporting Platform

The participants reported having a single and streamlined reporting system would encourage more ADR reporting:

I think just having a one stop shop.

CP1

One system for pharmacists to report.

CP5

#### User-friendly Reporting Systems (Integrated IT Systems Within the Clinical Workflow)

Having multiple reporting platforms and login passwords was considered a barrier by all participants. Integrating ADR reporting within their workflow was highly regarded.

If it was incorporated into the software. Like if we use Fred and you could just type it in there and then somehow feed its way through to TGA that'd be good.

CP7

If they want quick reporting, it should be built into the dispense program. If you bring the patients profile you click the drug that had a bad reaction and report the adverse drug reaction, then it pulls the information from the dispense software.

CP10

#### User-friendly Reporting Systems (Autopopulation Features Within the Dispensing Software)

Participants mentioned that future ADR reporting platforms should not only be integrated within the dispensing software, but the system design should be clinician focused:

It would be easy if you could just go through a patient's history and say, click on it and that would pre-populate with patients details from the dispensing software so you've automatically got the patient initials, details of the other medicines that they are taking, which may or may not be relevant, but the software could automatically do that.

CP8

I work with one i.e. [CP] that's a bit on the older side, and technology for her is not a strong point. If you're getting them to go between different programs, then for them to type in information when they're not quick at typing. That is [reporting] should be as easy as possible and I can't think of anything easier than it being built-in into the dispense system, so just right click the drug and then go and report adverse reaction.

CP2

#### User-friendly Reporting Systems (Efficient Reporting Forms)

Participants who had previously reported ADRs suggested having clear and succinct reporting forms, capturing the most pertinent information would facilitate ADR reporting:

Something that saves time and makes reporting more efficient than having to type out paragraphs and essays of what you're trying to report.

CP9

SafeVac was actually really easy to sign in. I can’t remember if I had to create an account, even if I did, it was surprisingly short. But I didn't feel like with SafeVac. I made a difference in any way because I just reported that he had a headache and then nausea, but I wanted to say that it was more prolonged, but it didn't allow me to say that, it was a multiple choice.

CP3

#### User-friendly Reporting Systems (Implementation of Artificial Intelligence to Detect ADRs Within Dispensing Systems)

Implementing reporting systems leveraging innovative technologies such AI was seen as an effective strategy to facilitate ADR reporting and reduce workload:

I think if there is something that we could do within the dispensing software that can expand to not just dispensing, if there's AI built into it, to detect any notes or clinical interventions that have ADRs in it and can pick up alerts.

CP3

Say your dispensing software. Fred dispense prompts you to fill out what allergy or reaction that happened, when it happened, pulls all the patient demographics info. If it then had a little thing, do you want to submit this to the TGA? and your able to go yep, bang, and then it would map and link all of the structured data and then send it off. That's good.

CP5

#### Implementing ADR Reporting Mobile Apps

Participants suggested mobile apps could facilitate ADR reporting by allowing CPs to report through their point of care digital tablets (Ipads) or integrating ADR reporting into existing systems (My Health Records app):

Even an app that people can report on their phones, sometimes in a pharmacy setting or their lunch break.

CP11

Maybe like an app or something, you can do it and pre-populates your information, like your name, that sort of stuff.

CP9

#### Consumer Follow-up and Clinician Feedback

Participants who previously reported an ADR suggested feedback from regulatory agencies could provide positive reinforcement and facilitate ADR reporting:

I think some feedback would be good because sometimes you got questions, right? Is this actually happening across Australia or globally?

CP11

Maybe now that this information is housed somewhere centrally [after ADR reporting], they can even contact you like after three months, six months or twelve months to see how your adverse drug reaction was.

CP7

## Discussion

### Principal Findings

The knowledge and perspectives of CPs in Australia regarding ADRs and ADR reporting practices have been quantitatively described by Li et al [9]. However, in this study, we conducted a qualitative analysis. To our knowledge, this is the first qualitative study in Australia that explores the perceptions of CPs regarding PV by using a theoretical framework that maps out the barriers to and enablers of ADR reporting and recommends digital intervention strategies.

In summary, this study demonstrated that lack of knowledge is a key driver of low awareness of PV or reporting systems, while workplace factors and lack of facilitating resources are key drivers of the lack of time to report by CPs in Australia. Developing multifaceted digital reporting systems within the pharmacist’s workflow can facilitate ADR reporting. A multifaceted digital reporting tool may use autopopulation features as well as an integrated ADR reporting and feedback system within the pharmacist dispensing interface.

### Lack of Knowledge as Driver of Low Awareness

The interview findings revealed a lack of awareness among the participants concerning ADR reporting systems and reporting of ADRs to Australian regulators, with all participants stating “Google” as their primary starting point. Furthermore, all CP participants (N=12) were unaware of the built-in adverse event recording module or feature of the GuildCare system, despite having used the system to conduct various clinical tasks; for example, distributing COVID-19 rapid antigen tests and recording immunizations, dose administration aids, or home medicine reviews. What is important to note is that the GuildCare ADR reporting feature has been available to CPs since 2014. A small number of CPs, that is, 33% (4/12), had previously reported an ADR directly to a regulatory authority. Among the 4 CPs, 2 (50%) had made an ADR report following immunization (ie, COVID-19 vaccines), and the other 2 (50%) CPs reported ADRs associated with medications. In Australia, it is important to note that vaccination providers, including CPs, are required to report vaccines administered to the Australian Immunisation Register, and jurisdictional legislation to report serious adverse events following immunization (AEFI) to local public health authorities may also apply [30,31]. By contrast, reporting ADRs associated with medicines (ie, excluding vaccines) is voluntary [11]. Therefore, jurisdictional legislation on vaccine reporting may have influenced the 2 CPs who reported ADRs after immunization. This possibility raises the potential for mandatory policy for ADR reporting as an intervention strategy that can be used in further research. In their submission to the 2015 TGA review of Australian Medicines and Medical Devices regulations, the Consumers Health Forum also argued for mandatory requirements for physicians and pharmacists to report ADRs [9].

A major theme identified within the data was the “reported” lack of sufficient education and training at a foundational level. Most respondents suggested increasing training and awareness to facilitate ADR reporting. All 12 participants recalled having little to no education on ADR reporting during the pharmacy curriculum and postgraduate internships, highlighting a key area for further exploration. Another theme was within the CPs workflow, where respondents suggested that their primary response to an ADR was to first ensure patient safety and then notify the prescriber. This was considered a satisfactory clinical workflow by all the respondents, and there was no mention of further activity such as reporting to the TGA. Nevertheless, to participate in PV, one must understand what PV is. The respondents subjectively used the terms “reporting drug allergies,” “incident reporting,” or “reporting drug interactions” within the context of ADR reporting, suggesting a lack of consensus on what constitutes ADRs. A lack of consensus on what constitutes an ADR among CPs suggests that considerations need to be given to include or provide more training on PV and ADR reporting such as within pharmacy curricula, prelicensure training, and continuing professional education workshops. This is consistent with previous studies showing that CPs have limited knowledge of PV, which may affect their ability to report ADRs in clinical practice [9,32].

### Work Environment or Resources as Drivers of Lack of Time

All CPs reported a lack of time as a major barrier to ADR reporting. Our findings are consistent with those from a previous quantitative survey, which suggested that nonreporting pharmacists were more likely to report lack of time as a barrier (*P*<.001) [9].

However, our qualitative analysis of the interviews allowed us to probe further into the theme of “lack of time” as a barrier to ADR reporting by discerning what CPs generally mean when they say, “lack of time to report.” According to our data, we were able to contextualize CPs’ reported “lack of time” as either a constraint to stop performing regular duties or perform an ADR reporting process, that is, from the consumer or patient contact to ADR report submission. The second context referred to the time to “completing a reporting form”; for example, the TGA or SafeVac web-based reporting webforms. Therefore, is “lack of time” simply a barrier to ADR reporting? Or, are there barriers limiting the time to report ADRs? Within the first context, “lack of time” is a dependent variable, influenced by external factors, such as the work environment and lack of support staff, while in the second context, it refers to the cumbersome reporting forms.

This brings us to the second point, regarding the lack of time to report. From our data, 33% (4/12) CPs had previously completed an ADR report, expressing frustrations around the “multitude of reports required to complete a single ADR reporting event, that is, the large amount of paperwork or administrative work involved” or the “number and types of questions asked, including the lack of appropriate response options available on the web-based reporting forms.” Nevertheless, all the participants in our study expressed challenges within their work environment as barriers, limiting their time to report. Many highlighted that there is not enough time to undertake their basic roles as a pharmacist and provide ADR reporting. If there were financial incentives, then this may support pharmacists by perhaps “buying” time for them to undertake this task in a busy pharmacy. However, while workplace resources were a challenge that could affect CPs’ capacity to report, the CPs who had previously reported an ADR (ie, 4/12, 33%) further stressed the need for succinct, centralized, and more user-friendly digital reporting forms. CPs who had previously reported an ADR associated their “lack of time to report” with a frustrating and inefficient ADR reporting form, affecting their time to complete the form.

These findings are consistent with a 2018 cross-sectional quantitative survey, in which CPs who had reported ADRs to the TGA did not perceive time as a barrier. The study noted that the underlying perspectives of individual pharmacists affected how they allocated time to perform ADR reporting as part of their professional practice [9]. Therefore, our findings suggest that clarity and a distinct understanding of what is meant by the phrase “lack of time to report” may be useful in designing more targeted intervention strategies. The first context relates to the organizational or workplace structures that may affect their time, and the second context relates to operational IT infrastructures that may affect time to complete ADR reports.

### Technology as a Facilitator of ADR Reporting

All CPs voiced the need to develop and integrate reporting systems using autopopulation features within pharmacy dispensing software, with a feedback loop. To our knowledge, 2 PV systems currently exist in Australian community pharmacies. In June 2014, a pharmacy software vendor GuildLink created GuildCare, an adverse events recording module linked to community pharmacy dispensing software and integrated directly into the TGA ADR web service [9]. Although integrating reporting systems into pharmacists dispensing software presents opportunities, it is important to note that not all community pharmacies in Australia make use of the same dispensing software or professional service program. Therefore, it is crucial for regulators or software vendors to develop uniform reporting or surveillance systems that can be integrated with available pharmacy dispensing programs [33].

One such systems integration was recently implemented for vaccine surveillance in Western Australia, in response to the COVID-19 pandemic; the vaccine safety surveillance system (SmartVax) was linked and integrated to a cloud-based community pharmacy software system (MedAdvisor) to measure AEFI reports [30]. MedAdvisor is a professional service data management system used by CPs that automatically reports immunizations administered directly to the Australian Immunization Register. SmartVax is a participant-centered active vaccine safety surveillance system that integrates with national surveillance networks in Australia [30]. Drug safety surveillance systems may be active or passive [34]. Passive surveillance systems provide opportunities for health care personnel to confidentially and voluntarily report ADRs, and active surveillance systematically monitors particular patient encounters to seek detailed information about adverse events that occur [30,34].

Therefore, implementing an automated active surveillance system that can link directly to all pharmacy medication systems may offer a simple and rapidly scalable option for drug safety surveillance with little impact on the pharmacist’s workload. These interventions may be further supported by the use of artificial intelligence that identifies possible ADRs and prompts the pharmacist when recording clinical data or dispensing medications. Furthermore, the use of mobile phone apps to facilitate ADR reporting was highly regarded by CPs. Comments on the use of mobile apps involved the ability to empower consumers to make their own reports. This may involve developing PV infrastructures within “My Health Record,” which is a personal electronic health record available to all Australians and integrating this directly to the TGA may also provide transparency to the major stakeholders within the digital health ecosystem. Mobile tools for active surveillance of AEFI via SMS text messaging have already been implemented in Australia [35]. Furthermore, apps for passive surveillance also exist in Europe and Canada and can provide the necessary benchmarks [36,37].

### Key Contributions and Recommendations

Besides addressing barriers to ADR reporting in Australia and suggesting interventional strategies to improve ADR reporting, the qualitative nature of this study provides context to the themes identified, such as “lack of time to report.” We theorize that it is not the end users (CPs) who need behavior change through more enticements or enforcement, but rather that the work practices and technologies that support their work need review or further investigations and altered where suitable.

On the basis of the findings of this study, our team posits 5 recommendations that may improve the rate of ADR underreporting by CPs in Australia. First, considerations need to be given to including more PV and ADR reporting into the pharmacy curricula at universities and licensure training and development of continuing education workshops to increase awareness and knowledge of ADR reporting. Second, work practices need to be revised to support the ADR reporting workflow, which may be supported by policies and procedures from organizations, such as the Pharmacy Guild of Australia or the Pharmaceutical Society of Australia. Third, to ensure the uptake and utility for clinical care, dispensing systems must act as a mechanism to document work and, with the use of autopopulation features, easily share information between the pharmacy and regulatory authorities; the addition of feedback loops may serve as positive reinforcement. Fourth, the use of artificial intelligence or integrating active ADR surveillance systems into existing medication management systems may be used to provide ADR alerts and warnings. Furthermore, surveillance systems can also be linked to the established national surveillance networks in Australia. Finally, consumers should be empowered to report ADRs via mobile phone apps. The development of these systems should consider all stakeholders within the health care ecosystem, ensuring transparency of information [38]. With the diffusion of new electronic prescription systems such as the Active Script List, considerations could be made to include ADR reporting within the system architecture. The use of existing systems, such as Australia’s national My Health Records, may also present an opportunity [39,40].

### Strength and Limitations

The strength of this study is its qualitative approach. This format allowed contextual insight into the participants’ responses, such as the commonly mentioned phrase “lack of time to report” and their perceived barriers and facilitators. We were also able to sample participants from different community pharmacy settings across rural and metropolitan areas. Barriers and facilitators emerged within the different domains of the TTF model and could offer insights into designing suitable improvements to optimize the quantity and quality of ADR reports. Implementing active and passive surveillance systems, as well as improving reporting systems, could enhance the exchange of safety data, prevent ADR-associated hospital admissions, and reduce health care expenditures. This could also be an essential step in making the data readily accessible for patient registries, research, or PV activities.

However, this study has some limitations that may affect the generalizability of our findings. The findings may be limited, as the sample was confined to a small number of participants working in Victoria, Australia. The CPs were selected by purposive sampling, which could have resulted in selection bias. All participants were from the geographic state of Victoria, and their responses may have been shaped by the organizational context for reporting ADRs within the jurisdiction. We also acknowledge that participants in this study self-selected to participate and may provide an element of responder bias, as more motivated individuals or those with a personal interest in PV or medication safety may have opted to participate. Some CPs may feel guilty for not reporting ADRs and therefore may have altered their responses to provide “socially desirable” responses about their perspectives toward ADR reporting. Although the sample may be seen as a limitation, there were varied opinions and from many who did not regularly report ADRs, suggesting that the strength of socially desirable bias may not be too strong. This study also focused its inquiry using a theoretical model, which may have limited the exploration of other important factors.

### Conclusions

This study highlights the individual and system-related barriers that influence ADR reporting among CPs practicing in Victoria, Australia. Classification of both barriers and facilitators using a theoretical framework could be effective in designing more tailored and suitable interventions targeting ADR underreporting. The results of the study demonstrated that lack of knowledge is a key driver of low awareness of PV or reporting systems among CPs, and work environment or resources are key drivers of the lack of time to report by CPs in Australia. Understanding the meaning and nature of “lack of time to report” may be useful to design more targeted intervention strategies, the first relating to the organizational workplace structure and the later, operational or IT infrastructure.

### Future Research

This study identifies several barriers and proposes different facilitators to overcome them as the first step. This study may encourage further research to evaluate the effectiveness of proposed intervention strategies. Future observational fieldwork should be conducted to observe physicians and pharmacists within their work settings. This approach will allow us to gain an understanding of the clinical workflow, work environment, and how ADRs are diagnosed, documented, and reported and barriers to reporting. In addition, research can be conducted to investigate and compare the perspectives of hospital pharmacists and CPs toward ADR reporting. Consumers’ perspectives and knowledge of ADRs could also provide insights into the barriers to and facilitators of consumer ADR presentations to CPs. It may be useful to explore policy changes including remuneration and mandatory reporting. Studies can be conducted with end users (CPs) and software vendors to discuss facilitators of ADR reporting including their involvement in the design of any future ADR reporting tools and their practical implementations.

As technology advances, risks and challenges may arise; therefore, further research may focus on developing standardized frameworks and guidelines that govern system integration and interoperability [41]. Finally, an ongoing evaluation of the effectiveness of existing and new ADR reporting technological systems may offer insights into the continual optimization of ADR reporting interventions.

## Appendix

Multimedia Appendix 1 Modified Monash category (2019).

## Abbreviations

ADR: adverse drug reaction
AEFI: adverse events following immunization
CP: community pharmacist
HCP: health care professional
MM: modified Monash
PV: pharmacovigilance
TGA: Therapeutic Goods Administration
TTF: Task-Technology Fit
