# Supplementary figures and images for: Barriers and Facilitators Influencing Real-time and Digital-Based Reporting of Adverse Drug Reactions by Community Pharmacists: Qualitative Study Using the Task-Technology Fit Framework
Source: Interact J Med Res. 2022 Oct 11;11(2):e40597. doi: 10.2196/40597 (PMC9597424; doi:10.2196/40597)

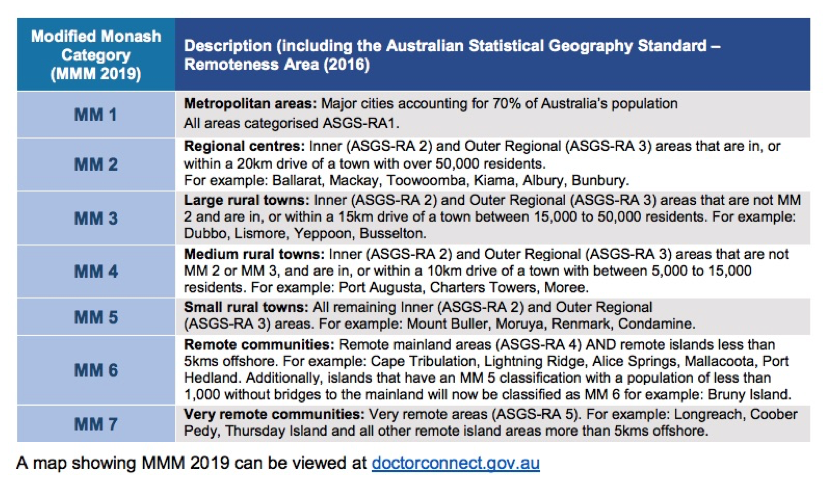

Supplement: Multimedia Appendix 1 [file ijmr_v11i2e40597_app1.png]
